# Fractionated stereotactic radiotherapy in the treatment of pituitary macroadenomas

**DOI:** 10.3747/co.v15i6.293

**Published:** 2008-12

**Authors:** H. Elhateer, T. Muanza, D. Roberge, R. Ruo, E. Eldebawy, C. Lambert, H. Patrocinio, G. Shenouda, L. Souhami

**Affiliations:** * Department of Radiation Oncology, McGill University Health Center, Montreal, QC; † Department of Medical Physics, McGill University Health Center, Montreal, QC

**Keywords:** Radiotherapy, fractionated stereotactic radiotherapy, macroadenoma, pituitary adenoma

## Abstract

**Background:**

The use of fractionated stereotactic radiotherapy (fsrt) has evolved with technical advances in noninvasive immobilization, radiation delivery, and image guidance. The application of fsrt to pituitary tumours is aimed at reducing toxicity through improved dose conformality and reduced treatment margins. The aim of the present paper is to report our own experience and to review the published data on fsrt for pituitary macroadenomas.

**Methods:**

Between September 2000 and October 2005, 13 patients with pituitary macroadenoma underwent fsrt at our institution. In 12 patients, radiotherapy treatment followed surgical resection (transsphenoidal resection in 8, frontal craniotomy in 3, and multiple transsphenoidal resections followed by craniotomy in 1). In 4 patients, the tumours were functional (2 adrenocorticotropic hormone–secreting, 1 prolactinoma, and 1 growth hormone–secreting); the tumours in the remaining patients were clinically non-secretory. Before radiation, 3 patients had panhypopituitarism, and 6 patients had visual field defects. All patients were treated with fsrt using non-coplanar micro–multileaf collimation portals. A median dose of 50.4 Gy (range: 45–60 Gy) was prescribed to the 76.9%–95.2% isodose surface and delivered in 1.8-Gy fractions. The median planning target volume (gross tumour plus 3 mm) was 33.5 cm^3^ (range: 3.2–75 cm^3^).

**Results:**

After a median follow-up of 24 months (range: 6–60 months), local control was 100%. One patient achieved clinical complete response. Treatment was well tolerated acutely for all patients. Neither radiation-induced optic neuropathy nor any radiation-related endocrine dysfunction was observed in our patients.

**Conclusions:**

In accordance with published series, we found fsrt to be safe and effective in the management of large pituitary macroadenomas.

## 1. INTRODUCTION

Pituitary tumours account for 10%–15% of primary intracranial neoplasms. “Macroadenoma” refers to a tumour more than 10 mm in diameter^1^. Typically, these tumours extend superiorly into the suprasellar cistern or laterally into the cavernous sinuses. In many cases, despite early benefit from surgical debulking, long-term cure from transsphenoidal surgery alone remains elusive. Radiotherapy is frequently used to treat residual or recurrent pituitary adenomas^2^. In retrospective series, immediate postoperative radiotherapy has clearly led to significant improvements in long-term local control. Nevertheless, concerns regarding potential late complications such as brain necrosis and optic nerve injury have limited or delayed the use of radiotherapy^3^,^4^. Classically, simple three-dimensional conformal plans (often a 3-field technique) were used to treat these central lesions, resulting in significant dose delivery to the temporal lobes and the optic apparatus.

With advances in noninvasive immobilization techniques, sophisticated planning systems, and image guidance, fractionated stereotactic radiotherapy (fsrt) has supplanted three-dimensional conformal radiotherapy in the treatment of macroadenomas. The fsrt technique combines the similar dose conformality, precise dose delivery, and steep dose falloff outside the target volume of stereotactic radiosurgery with the radiobiologic advantages of dose fractionation. Fractionation safely treats larger tumour volumes intimate to critical structures such as the optic apparatus^3^,^5^,^6^. Here, we report our experience of fsrt in a series of 13 patients whose pituitary macroadenomas were in close relationship with the optic chiasm or the cavernous sinus, or both.

## 2. PATIENTS AND METHODS

Between September 2000 and October 2005, 13 patients (9 men, 4 women) with pituitary macroadenoma were treated with fsrt at the Montreal General Hospital. These patients had a median age of 56 years (range: 30–80 years). In 9 patients, the tumours were non-secreting; tumours in the other 4 patients were functional (2 causing Cushing disease, 1 causing acromegaly, and 1 being a prolactinoma). Surgical resection was the initial treatment in 12 patients: 8 had transsphenoidal resections, 3 had frontal craniotomies, and 1 had multiple transsphenoidal resections followed by craniotomy.

The indications for fsrt were postoperative residual disease in 5 patients (38.4%) at a median of 3.2 months from surgery (range: 1.2–7.4 months), recurrent disease in 7 patients (53.8%) at a median of 12.4 months from surgery (range: 5.2–53.3 months), and primary therapy in 1 patient. Table I summarizes patient characteristics. All patients underwent complete clinical evaluation before fsrt, including consultations in ophthalmology and endocrinology.

A pretreatment 2-mm global T1 contrast-enhanced magnetic resonance imaging (mri) study was obtained for all patients and co-registered to a 3-mm treatment planning computed tomography scan. Tumour and normal structures were contoured, and a 3-mm margin was then added to the gross tumour volume to generate the planning target volume (ptv). Treatment plans were typically designed using 4–6 static 6-MV non-coplanar micro–multileaf collimation portals (Brainscan 5.21: BrainLAB AG, Heimstetten Germany). Typically, a dose of 50.4 Gy was prescribed to a median isodose surface of 90.1% (range: 76.9%–95.2%) in 1.8-Gy fractions over 5.5 weeks. A different regimen was used to treat 2 patients: one patient was treated to 45 Gy; the other, whose tumour had malignant features, received 60 Gy. Patients were immobilized using a noninvasive thermoplastic mask system (BrainLAB AG), with the patient supine in neutral head position.

Patients were followed regularly after treatment, 3 times during the first year, and then every 6 months thereafter. Follow-up included clinical assessment, mri studies, and hormonal assays. When warranted, visual field and formalized visual acuity testing was performed.

## 3. RESULTS

The median ptv for all 13 patients was 33.5 cm^3^ (range: 3.1–75 cm^3^). All patients had extrasellar tumour extension. In 2 patients (15.3%), extension reached the suprasellar region, and in 11 patients (84.6%), it reached the cavernous sinus.

**FIGURE 1:**
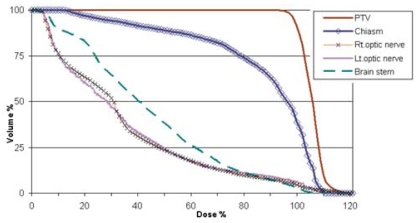
Cumulative representation of the average dose–volume histogram of 13 patients with pituitary macroadenoma. ptv = planning target volume; Rt = right; Lt = left.

The use of the stereotactic technique provided appropriate conformation of the prescribed dose to the ptv with a median planning isodose–tumour volume conformity index of 1.4 (range: 1.2–1.8). Between 93.3% and 99.1% (median: 97%) of the ptv was covered by the prescription isodose. In all patients, a portion of the optic chiasm and the optic nerves was spared from receiving the prescribed radiation dose. Because of proximity to the target, a small portion of the optic apparatus could have received a dose equal to or slightly higher than the prescribed dose. Hot spots of up to 110% of the prescribed dose were on occasion delivered to 5%–16% of the volume of the optic chiasm or the optic nerves. Figure 1 shows an averaged dose–volume histogram for all patients, and Table II summarizes the treatment parameters.

Treatments were well tolerated. No acute effects at grade 2 or higher were recorded, and to date, no late toxicity has been observed. All patients completed the prescribed radiation dose without treatment interruption.

**FIGURE 2:**
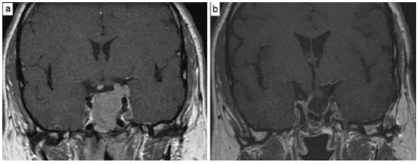
Magnetic resonance imaging (mri) in a 38-year-old man with adrenocorticotropic hormone–secreting adenoma. (a) Pretreatment image shows a large postoperative residual tumour. (b) Follow-up image 4 years after fractionated stereotactic radiotherapy shows complete response.

At a median follow-up of 27 months (range: 10–82 months), the local control rate—defined as freedom from radiologic disease progression—was 100%. In 2 patients (15.4%), an objective radiologic response was achieved. Complete tumour resolution was achieved in 1 patient with a large residual adrenocorticotropic hormone (acth)–secreting macroadenoma. His follow-up mri study revealed no radiologic evidence of disease 4 years after fsrt (Figure 2). Another patient who received primary fsrt for a growth hormone–secreting adenoma achieved a partial (50% or better) reduction in the original tumour volume. Radiologic findings were stable in the other 11 patients.

Of the 4 patients with functional macroadenomas, only 1 patient with an acth-secreting macroadenoma showed hormone level normalization from the pretreatment elevated level. This response was associated with a complete radiologic response (Figure 2). No significant change in hormone level was observed in the other 3 patients.

Pre-existing panhypopituitarism in 3 patients (23%) required continued hormone replacement therapy during and after fsrt. At last endocrinology follow-up, no manifestations of pituitary insufficiency were observed in the other 6 patients who presented with normal pituitary function.

Before the fsrt, 5 patients had an objective visual field defect, and 1 patient had occulomotor nerve injury. These original neurologic deficits were not changed after radiation therapy. Furthermore, no abnormalities were seen in the follow-up visual assessments of the remaining patients.

## 4. DISCUSSION

Surgical resection remains the initial treatment of choice for symptomatic non-prolactin-secreting pituitary macroadenomas^7^,^8^. Surgery offers the advantages of pathologic confirmation, immediate decompression of the optic apparatus, and rapid reduction of pathologic hormone secretion^7^,^9^. Long-term tumour control rates after surgery alone range between 50% and 80%^10^. However, a complete resection is achievable in only 44%–84% of patients with pituitary macroadenoma 11. The risk of local recurrence after subtotal resection is 33%–80%. Postoperative fractionated radiation is highly effective, leading to a 15-year local control rate of 95% 9,12–14.

Despite well-established long-term tumour control rates for adjuvant radiation therapy in pituitary macroadenoma, use of radiation is limited or frequently delayed because of concerns over potential late complications involving the optic apparatus, the pituitary gland, or the brain parenchyma 13,14. The most common complication after postoperative radiation therapy is pituitary hormone deficiency, whose reported rate ranges between 30% and 50% 15. The risk of developing hormone insufficiency is persistent for up to more than 15 years after radiotherapy. Brada *et al.* reported a need for hormonal replacement therapy in 30% and 50% of patients 10 and 19 years after radiotherapy 16. Optic neuropathy is a serious complication, but it is rare after fractionated radiotherapy to doses of 45–54 Gy; Parsons *et al.* reported no optic nerve injuries in 106 optic nerves that received a total radiation dose of less than 59 Gy 17. The reported risk of optic neuropathy after fractionated radiotherapy for pituitary adenomas lies between 1% and 5% 3. Radiation-induced secondary intracranial tumours in the form of sarcomas, meningiomas, and gliomas were also reported with cumulative risks of 1.3% and 1.9% after 10 and 20 years respectively 18. Other reported complications include brain necrosis, cerebrovascular disease, and neurocognitive dysfunction, although these risks are not well defined nor is the contribution of radiation to these events 3,17,18.

**FIGURE 3:**
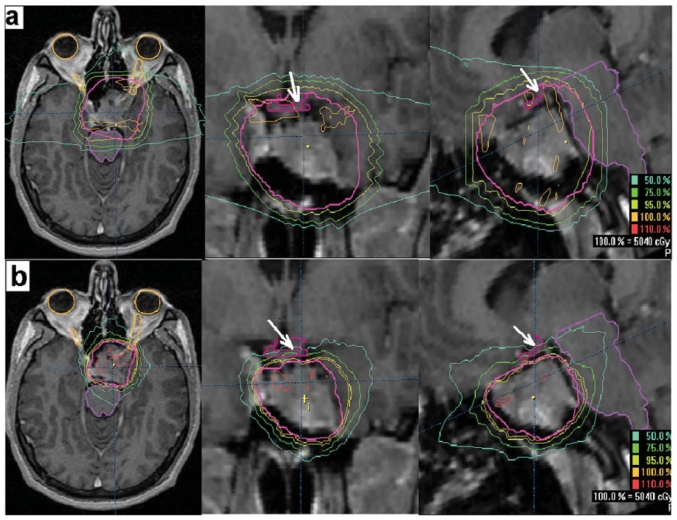
Axial, coronal, and sagittal magnetic resonance imaging slices with isodose distributions from (a) conventional 3-field technique, showing coverage of the planning target volume (ptv) and optic chiasm by the 95% isodose surface; and (b) 5-field fractionated stereotactic radiotherapy technique showing coverage of the ptv by the 100% isodose surface, while keeping the optic chiasm (arrow) outside the 75% isodose surface.

The foregoing risk estimates are derived from series in which less-conformal techniques were used to deliver radiation therapy. The image-guided stereotactic techniques currently available allow for more precise targeting, more-conformal dose distributions, and steeper dose falloff beyond the target volume, thereby reducing normal-tissue exposure and, consequently, expected late toxicities. Figure 3 depicts the dosimetric difference between conventional 3-field and fsrt plans. Not only does the latter plan provide better target coverage (the ptv is entirely within the prescription isodose), but also significant sparing of the optic chiasm.

Stereotactic radiosurgery (srs), either Gamma Knife (Elekta, Stockholm, Sweden) or linear accelerator–based, has frequently been used in the treatment of pituitary adenoma, with crude local control rates ranging from 90% to 100% and widely variable hormonal cure rates ranging from 0% to 100% 3,4. In a recent retrospective comparison, no statistically significant differences were observed between srs and conventionally fractionated conformal radiotherapy in terms of objective response rate (81.8% at 4 years) and local control rate (97% at 4 years). However, a trend toward a higher complete hormonal response rate was observed in the srs group (43.8% vs. 36.4%), with a shorter median time to complete hormonal remission (26 months in the srs group and 63 months in the fractionated radiotherapy group). No patients developed new visual deterioration, and of 11 patients who required post-treatment hormone replacement therapy, only 1 was in the srs group 19.

Reports from retrospective trials of srs specifically addressing pituitary macroadenoma show similar results 20,21, although in one series, macroadenomas tended to have worse local control. Local failures were also more common in cases in which the tumour extended into the cavernous sinus 20.

The limitation of srs is radiation-induced optic neuropathy after a single large radiation dose. The risk is low with doses below 10 Gy, but it increases to 26.7% at doses of between 10 Gy and 15 Gy. Doses higher than 15 Gy are associated with a markedly increased risk that may reach 77.8% 21. The current consensus is to limit radiosurgery to smaller adenomas removed from the optic apparatus (usually 3–5 mm) and to keep the dose to the optic apparatus below 8–10 Gy 3. Whether the outcomes for small tumours are different for srs and for fractionated radiotherapy remains controversial. Treatment selection is often guided by institutional preference and treatment device rather than by clinical evidence.

Currently, the use of fsrt is growing. The technique combines the precise treatment delivery of srs with the radiobiologic advantage of dose fractionation, making it the preferred technique for the treatment of large tumours or tumours closely situated to the optic apparatus. In one of the earliest retrospective reports on fsrt, Mitsumori *et al.* 22 reported the results of 30 patients treated with fsrt to a total dose of 45 Gy in 25 fractions, compared with 18 patients treated with srs to a dose ranging from 10 Gy to 18 Gy. The 3-year tumour control rate was not significantly different (100% for srs and 85.3% for fsrt) The median tumour volume in the srs group was much smaller, at 1.9 cm^3^ as compared with 5.7 cm^3^ in the fsrt group. The time to hormone normalization was shorter among srs patients, at 8.5 months as compared with 18 months for fsrt patients. The authors reported no radiation-induced visual complications in either group. The rates of hormone deficiency after radiotherapy were not significantly different (23% after srs vs. 20% after fsrt). The only significant difference noted was in the rate of radiation-induced brain necrosis, which was 27.8% in the srs group as compared with 0% in the fsrt group (*p* = 0.02). The authors recommended the use of fsrt for pituitary adenoma whenever possible.

Table III summarizes the published data on fsrt for the treatment of pituitary adenoma. Although the proportions of macroadenoma in these series were not specified, most series had to have included patients with larger tumours, given the median tumour volume range of 4.1–30.2 cm^3^.

Most studies have short follow-up periods. The reported local control rate ranged from 85% to 100%. The reported hormonal control rates are difficult to interpret because the definition of hormonal control varied with the study and because the number of patients with secretory adenomas was small. The same uncertainty applies to the reported visual complications, which ranged from 0% to 7%. The dose to the optic chiasm and the optic nerves, the types of visual complications, and the methods used to diagnose those complications were poorly reported in most studies.

In the current series, the median tumour volume was 13.5 cm^3^ (range: 1.1–47.6 cm^3^), with most tumours (84.5%) extending into the cavernous sinus. In terms of local control, our early results with macroadenomas are encouraging and comparable to those reported in the studies described earlier. The data are insufficient to draw conclusions with regard to hormonal response, given our relatively small and heterogeneous group of patients with secreting adenomas.

## 5. CONCLUSIONS

Although studies with long-term follow-up are lacking, fsrt appears to combine effective tumour control with a low incidence of radiation-related morbidity in patients treated for pituitary macroadenoma.

## Figures and Tables

**TABLE I:** Patient characteristics

*Characteristic*	n *(%)*
Age (years)	
Median	56
Range	30–80
Sex	
Male	9 (69)
Female	4 (31)
Tumour type	
Non-functioning	9 (69)
Prolactinoma	1 (8)
gh-secreting	1 (8)
acth-secreting	2 (15)
Indication for radiotherapy	
Postoperative	5 (38)
Disease progression	7 (54)
Definitive	1 (8)
Tumour volume (cm^3^)	
Median	13.5
Range	1.1–47.6
Pretreatment tumour extension	
Suprasellar	2 (15)
Parasellar	11 (85)
Pretreatment hormonal status	
Normal	6 (46)
Hypopituitarism	3 (23)
Oversecretion	4 (31)
Pretreatment visual status
Intact	3 (23)
Subjective visual symptom	4 (31)
Objective visual field defect	5 (38)
Oculomotor nerve injury	1 (8)
Presenting symptom	
Visual	9 (69)
Hormonal	3 (23)
Headache	1 (8)

gh = growth hormone; acth = adrenocorticotropic hormone.

**TABLE II:** Treatment parameters

Parameter	Median^a^	Range^a^
Planning target volume (ptv [cm^3^])	33.5	3.1–75
Total dose (Gy)	50.4	45–60
Fractions (*n*)	28	23–33
Fields (*n*)	5	4–6
Prescription isodose surface (%)	90.1	76.9–95.2
Conformity index	1.4	1.2–1.8
Dose covering 99% of the ptv (%)	98	94–100
Maximum dose to the chiasm (Gy)	52.9	44.3–55.5
Mean dose to the chiasm (Gy)	42.6	18.6–49
Maximum dose to optic nerves (Gy)	51.2	17–55.4
Mean dose to the left optic nerve (Gy)	15.3	5–28.5

a Average calculation for all patients.

**TABLE III:** Summary of series on fractionated stereotactic radiotherapy

Study	Patients (*n*) with					Control (%)		Toxicity (%)	
	Non-functioning tumour	Functioning tumour	Median tumour volume (cm^3^)	Median dose (Gy)	Median follow-up (mo.)	Local	Hormonal	Visual	Hormonal
Mitsumori *et al.,* 1998 22	12	18	5.73	45	34	85.3	23	0	20
Milker–Zabel *et al.,* 2001 23	42	20^a^	30.2	50.4–52.2	38.7	93	25	7	4.8
Milker–Zabel *et al.,* 2004 24	0	20^a^	26.2	52.2	59.8	100	80 a	5	10
Cañón Rodríguez *et al.,* 2005	24	32	8.9	54	51	92	na	3.5	46
Colin *et al.,* 2005 26	63	47	4.2	50.4	82	99	42.5	0	36.7
Paek *et al.,* 2005 5	68	0	6.2	46–50.4	30	98^b^	—	2.9	6
Voduc *et al.,* 2005 27	19	17	4.1	50.4	19.1	100	30	0	20
Minniti *et al.,* 2006 28	67	25	12.2	54	32	98^b^	36	1	22
Selch *et al.,* 2006 29	33	6	10.5	48.6	32	100	0	0	19
McClelland *et al.,* 2007 30	7	5	21.8	50.4	22.5	100	NA	0	0
Present study	9	4	13.5	50.4	27	100	25	0	0

a Twelve patients replicated between these two studies.

b Actuarial 5-year progression-free survival.

na = not assessed.
